# Drug-drug interactions in polypharmacy patients: The impact of renal impairment

**DOI:** 10.1016/j.crphar.2021.100020

**Published:** 2021-03-29

**Authors:** Bianca Papotti, Cinzia Marchi, Maria Pia Adorni, Francesco Potì

**Affiliations:** a University of Parma, Department of Food and Drug, 43124, Parma, Italy; b University of Parma, Department of Medicine and Surgery – Unit of Neurosciences, 43125, Parma, Italy

**Keywords:** CKD, Drug-drug interactions, Polypharmacy, Kidney failure

## Abstract

Chronic kidney disease (CKD) is a long-term condition characterized by a gradual loss of kidney functions, usually accompanied by other comorbidities including cardiovascular diseases (hypertension, heart failure and stroke) and diabetes mellitus. Therefore, multiple pharmacological prescriptions are very common in these patients. Epidemiological and clinical observations have shown that polypharmacy may increase the probability of adverse drug reactions (ADRs), possibly through a higher risk of drug-drug interactions (DDIs). Renal impairment may further worsen this scenario by affecting the physiological and biochemical pathways underlying pharmacokinetics and ultimately modifying the pharmacodynamic responses. It has been estimated that the prevalence of DDIs in CKD patients ranged between 56.9% and 89.1%, accounting for a significant increase in healthcare costs, length and frequency of hospitalization, with a detrimental impact on health and quality of life of these patients. Despite these recognized high-risk conditions, scientific literature released on this topic is still limited. Basing on the most commonly prescribed therapies in patients with CKD, the present short review summarizes the current state of knowledge of the putative DDIs occurring in CKD patients undergoing polytherapy. The most relevant underlying mechanisms and their clinical significance are also debated.

## 1 Introduction

The identification and characterization of chronic kidney disease (CKD) have developed progressively over time. Current international guidelines identified CKD as a condition of decreased kidney function described as glomerular filtration rate (GFR) ​< ​60 ​ml/min per 1.73 ​m^2^, or markers of kidney damage, or both, for at least 3 months of duration. Markers of kidney damage included albuminuria, urinary sediment abnormality, electrolyte or other abnormality due to tubular disorder and histological structural abnormalities (K/clinical practice, 2002). CKD is classified into five stages of increasing severity based on GFR values. When the end stage kidney disease (ESKD) is established, the kidney replacement therapy, represented by dialysis or kidney transplantation, is the only therapeutic option (K/clinical practice, 2002).

In 2017, there were 697.5 million cases of CKD worldwide, one third of them living in two countries: China (132,3 million cases) and India (115,1 million cases). Bangladesh, Brazil, Indonesia, Japan, Mexico, Nigeria, Pakistan, Russia, USA, and Vietnam accounted more than 10 million cases of CKD each (Bikbov et al., 2020).

CKD is a multifactorial disorder and comorbidities are often frequent and present from the early stages of the disease. Non-modifiable risk factors include age, gender, race, diabetes, and genetic causes, while modifiable risk factors include hypertension, proteinuria, and metabolic factors (Levey and Coresh, 2012). Genetic determined CKD only minimally accounts for all CKD patients.

As CKD patients exhibit a high number of comorbidities, including underlying diseases and deleterious consequences of impaired kidney function, multiple medications are needed.

Polypharmacy has been defined as the concomitant use of five or more drugs per day by a single person (Fincke et al., 2005; Morin et al., 2018). Combination of prescriptions and over the counter (OTC) drugs increases the probability of adverse drug reactions (ADRs) and drug-drug interactions (DDIs), which are leading causes of an increased risk of hospitalization and death (Morin et al., 2018). The prevalence of DDIs in CKD patients has been estimated between 56.9% and 89.1% (Rama et al., 2012; Sgnaolin et al., 2014; Marquito et al., 2014; Hegde et al., 2015; Al-Ramahi et al., 2016; Saleem et al., 2017; Fasipe et al., 2018), probably because polypharmacy itself is necessary for the management of this complex condition. Laville et al. demonstrated that polytherapy increases the odds of hospitalization and death, and receiving one or more inappropriate treatment further increase the risk for adverse drug reactions (Lazarou et al., 1998; Laville et al., 2018). Fokter and colleagues also reported an association between the number of drugs on hospital admission and the presence of kidney failure, without any further association with age, sex, liver failure and urgency of admission (Fokter et al., 2010).

Importantly, variations in pharmacodynamic (PD) and pharmacokinetic (PK) parameters in patients with renal impairment further aggravate this pathological condition (Matzke et al., 2011). In this respect, it is well-known that the kidneys play an important role in the handling of drugs, most importantly in their excretion. A consequence of CKD on PK is a reduction in renal clearance due to a decrease in the GFR. The impact of renal impairment on PK is, however, not limited to a decreased elimination of drugs excreted by the kidneys. CKD is associated with multiple physiological changes and may therefore influence extrarenal PK processes, such as drugs absorption, distribution and metabolism, which may increase the risk of toxicity (Lea-Henry et al., 2018). Consequently, patients with impaired kidney function are more at risk of altered drug exposure or toxic effects than individuals with normal kidney function (Bates et al., 1999). In this context, studies in experimental models of CKD have demonstrated an altered expression and/or activity of both intestinal and hepatic drug transporters impacting on intestinal absorption and hepatic uptake, respectively, and eventually on drug metabolism. The proposed mechanisms for the impairment of drug metabolism in CKD include alterations in gene transcription and protein translation, reduced CYP expression due to inhibition of hemoprotein biosynthesis and/or increased enzyme degradation, depletion of cofactors (e.g., supply of nicotinamide adenine dinucleotide phosphate (NADPH)), and direct competitive inhibition of CYP enzyme by circulating uremic constituents (Nolin et al., 2008). In this regard, Nolin and colleagues have summarized experimental studies which demonstrated reduced expression of CYP genes and gene products (i.e. reduced mRNA and protein, or reduced protein with no change in mRNA) in several animal models of CKD (Nolin et al., 2008).

In the present short review, we summarize and critically examine the current state of knowledge on the most prevalent drug interactions occurring in CKD patients undergoing polytherapy, the most relevant underlying mechanisms and the clinical significance of the putative DDI.

## 2 Patterns of medications used in CKD patients

Individuals with CKD are usually affected by a high number of comorbidities, including underlying diseases as well as consequences of reduced renal functionality, such as hypertension, diabetes, cardiovascular disease (CVD), anemia and bone and mineral disease (Gyebi et al., 2012; Johnson et al., 2013; Hostetter et al., 2001). These conditions require multiple medication to ameliorate patients’ symptoms and slow the progression of the disease, increasing, however, the risk for the development of drug interactions. Few published reports in CKD patients evaluated the prevalent medication pattern looking at the specific drug classes; the most relevant are summarized in Table 1. However, the available studies are not easily comparable to each other due the heterogeneity in the study design, the study population and the equation used to estimate kidney function.

**Table 1:** Patterns of medications among patients with CKD.

STUDY	COUNTRY	NUMBER OF PATIENTS	AGE (years; mean ​± ​SD)	ESKD PATIENTS (%)	NUMBER OF MEDICATIONS (Mean ​± ​SD)	COMORBIDITIES	MOST COMMONLY PRESCRIBED DRUGS
Bailie et al. (2005)	USA	619	60.6 ​± ​16.0	18.74%	8 ​± ​4	Diabetes (37%); Hypertension (90%) and CAD (28%)	Calcium channel blockers (52%), β-blockers (46%), ACE inhibitors (44%), Aspirin (37%), Erythropoietin (20%), HMGCoA reductase inhibitors (16%), Intravenous iron (13%), Angiotensin receptor blockers (13%)

Marquito et al. (2013)	Brazil	558	69.4% elderly (NS)	6.6%	5.6 ​± ​3.2	Hypertension (68.5%); Diabetes mellitus (32%); Coronary disease (6.63%); Heart failure (5.2%)	Furosemide (8.4%), Simvastatin (7.1%), Losartan (7.1%), Aspirin (5.2%), Captopril (4.7%), Hydrochlorothiazide (4.7%), Omeprazole (4.5%), Enalapril (4.1%), Amlodipine besylate (3.3%), and Nifedipine (3.1%)

Al-Ramahi et al. (2016)	Palestina	275	50.67 ​± ​15.93	100%	7.87 ​± ​2.44	Hypertension (78.5%); Diabetes mellitus (42.5%); Gout (9.5%); Myocardial infarction (8.4%); Hyperlipidemia (6.2%); Congestive heart failure (5.8%)	CaCO_3_ (77.1%), α-Calcidol (73.8%), Folic acid (65.5%), Aspirin (54.9%), Amlodipine (49.5%)

Fasipe et al., 2018, Fasipe et al., 2017a, Fasipe et al., 2017b	Nigeria	123	53.81 ​± ​16.03	69.9%	10.28 ​± ​3.85	Hypertension (83.7%); Diabetes mellitus (31.7%); Obesity (19.5%); Heart failure (8.9%); Obstructive uropathy (6.5%); HIV (5.7%); Stroke (4%); PKD (4%), HBV (4%)	Furosemide (71.6%), Lisinopril (52.9%), CaCO_3_ (51.2%), α-Calcidol (50.4%), Erythropoietin (49.6%), Intravenous Iron Sucrose (48.8%), Amlodipine (45.5%), Hydrochlorothiazide (43.1%), Folic acid (43.1%), OFS (40.7%)

Laville et al. (2018)	France	3033	69	41% stage 4 4% stage 5	8	Hypertension (91%); Diabetes (43%); Dyslipidemia (75%); CVD (54%); AKI (24%)	Antihypertensive agents (94%), Lipid-modifying agents (63%), Antithrombotic agents (60%), Antidiabetic agents (36%), Drugs for acid-related gastrointestinal disorders (34%), Anti-gout preparations (34%), Analgesics (24%), Psycholeptics (17%), Mineral supplements (16%)

Secora et al. (2018)	USA	6392	76.3 ​± ​5.2	G5 0.2% G4 1.4%	6.1 ​± ​3.5 medications; 2.3 ​± ​2.2 vitamins or supplements.	Hypertension (69.8%); Diabetes (32.4%); Heart failure (18.6%); CVD (14.7%); Myocardial infarction (7.6%)	Antihypertensive agents (75.4%; mostly β-blockers), Aspirin-containing medications (59.4%), Lipid-modifying agents (55.6%), β-blockers (33.5%), NSAID-containing (27.3%) Diabetes medications (19.9%)

Schmidt et al. (2019)	Germany	5217	18–74	G4 and G5: 8.83%	8	Diabetes mellitus; Heart failure; Hypertension; CHD; Cerebrovascular disease; Peripheral vascular disease; CVD; Dyslipidemia; Anemia	β-blockers and ACE inhibitors (e.g. ramipril,31.7%), lipid-lowering drugs (e.g. simvastatin 38.4%), diuretics (e.g. torasemide 28.3%, allopurinol 31%; hydrochlorothiazide 26.6%), platelet aggregation inhibitors (e.g. acetylsalicylic acid 32.6%) and urate-lowering therapy (e.g. allopurinol 31%). Vitamin D supplements (31%), supplementary iron (5%), Folic acid (2%), homeopathic agents (3–5%).

Santos-Díaz et al. (2020)	Spain	122	77.1 ​± ​10.4	4.5%	8.6 ​± ​3.4	Hypertension (52%); Diabetes mellitus (25%); Dyslipidemia (33%); Anemia (13%); Hyperuricemia (11%)	Omeprazole (30.6%), acetaminophen (30.6%), salicylic acid (26.1%), bisoprolol (25.2%), furosemide (22.5%), and allopurinol (21.6%)

Subeesh et al. (2020)	India	160	50.08 ​± ​15.32	_	9.16 ​± ​3.01	Hypertension (100%); Diabetes mellitus (29.4%); Anemia (11.2%); IHD (9.4%); Hypotiroidism (4.4%)	Diuretics (77.50%), α-agonists (32.50%), α-blockers (25.62%), and β-blockers (11.87%) and insulin (25.62%)

ACE: Angiotensin–Converting Enzyme; AKI: acute kidney injury; CAD: coronary artery disease; CHD: coronary heart disease; CVD: cardiovascular disease; HBV: hepatitis B virus; HIV: human immunodeficiency virus; HMGCoA: 3-hydroxy-3-methyl-glutaryl-coenzyme A; IHD: ischemic heart disease; NSAID: Nonsteroidal anti-inflammatory drugs; PKD: polycystic kidney disease.

**Table 2:** Selection of clinical evidences reporting the burden of polypharmacy, prevalence and severity of potential DDI among patients with CKD.

STUDY	COUNTRY	TYPE OF STUDY	NUMBER OF PATIENTS	AGE (years; mean ​± ​SD)	ESKD PATIENTS (%)	NUMBER OF MEDICATIONS (Mean ​± ​SD)	POLYTHERAPY (%)	MOST FREQUENT DRUGS	MOST FREQUENT DDI	SEVERE INTERACTIONS (%)
Rama et al. (2012)	India	Prospective, observational study	205	48.58 ​± ​16.23	68.48%	12.08 ​± ​6.3	NA	NA	Ascorbic acid – Cyanocobalamine (12.45%) Clonidine – Metoprolol (3.8%) Amlodipine – Metoprolol (3.38%) Insulin – Metoprolol (2.95%)	20%

Marquito et al. (2014)	Brazil	Cross-sectional, observational study	558	69.4% elderly (NS)	6.6%	5.6 ​± ​3.2	NA	Furosemide (8.4%), Simvastatin (7.1%) Losartan (7.1%) Aspirin (5.2%) Captopril (4.7%)	Furosemide – Aspirin (7.8%) Enalapril – Furosemide (5.9%) Captopril – Furosemide (5.1% Enalapril – Losartan (3.7%) Allopurinol – Captopril (1.8%)	16.8%

Sgnaolin et al. (2014)	Brazil	Cross-sectional, observational study	65	59.1 ​± ​14.7	100%	6.3 ​± ​3.1	87.7%	CaCO_3_ (84.6%) Erythropoietin (72.3%) Sodium citrate (60%) Omeprazole (29.2%) Calcitriole (27.7%)	Atenolol - CaCO_3_ (8%) CaCO_3_ – OFS (8%) CaCO_3_ – Ticlopidine (6.3%) Enalapril – Erythropoietin (4.5%) Amiodarone – Prednisone (3.6%)	27.6%

Hegde et al. (2015)	India	Cross-sectional, observational study	120	58.53 ​± ​8.38	NS	9.4 ​± ​3.9	NA	NA	Sodium bicarbonate – OFS (8.9%) CaCO_3_ – OFS (5.5%) Aspirin – Carvedilol (5.5%) Sodium bicarbonate – Allopurinol (5.5%) Pantoprazole – OFS (4.79%)	16.41%

Al-Ramahi et al. (2016)	Palestina	Observational – retrospective cohort study	275	50.67 ​± ​15.93	100%	7.87 ​± ​2.44	90.5%	CaCO_3_ (77.1%) Alpha Calcidol (73.8%) OFS (65.5%) Folic Acid (65.5%) Aspirin (54.9%)	CaCO_3_ – Amlodipine (12.3% CaCO_3_ – Aspirin (8.2%) Aspirin – Furosemide (7.9%) Aspirin – Enoxaparin (4.3%) Aspirin – Insulin (4.19%)	8.39%

Fasipe et al., 2018, Fasipe et al., 2017a, Fasipe et al., 2017b	Nigeria	Retrospective study	123	53.81 ​± ​16.03	69.9%	10.28 ​± ​3.85	85.4%	Furosemide (71.6%) Heparin (54.47%) Lisinopril (52.9%) CaCO_3_ (51.2%) Alpha Calcidol (50.4%)	CaCO_3_ – OFS (9.94%) Folic acid – Furosemide (3.4%) Alpha Calcidol - CaCO_3_ (3.24%) OFS ​+ ​Vitamin E (3.03%) CaCO_3_ – Furosemide (2.65%)	2.7%

Saleem et al. (2017)	Pakistan	Retrospective study	209	38.34 ​± ​16.82	74.2%	NA	78%	NA	OFS – Omeprazole (5.8%) Calcium/Vitamin D – Ciprofloxacin (4.8%) Captopril – Furosemide (4.1%) Calcium gluconate – Ceftriaxone (3.6%) Ciprofloxacin – OFS (2.9%)	27.8%

Adibe et al. (2017)	Nigeria	Retrospective study	169	51.03 ​± ​14.9	28.4%	6.15 ​± ​1.96	NA	Furosemide (11.48%) Lisinopril (8.85%) Amlodipine (7.3%) Ranitidine (6.81%) Hydrochlorothiazide (6.61%)	Lisinopril – Furosemide (9.06%) Furosemide - CaCO_3_ (7.22) CaCO_3_ – Lisinopril (6.11%) Aspirin – Furosemide (4.58%) Furosemide - Hydrochlorothiazide (4.44%)	3.87%

Okoro and Farate (2019)	Nigeria	Cross-sectional study	201	49.5 ​± ​14.5	70%	5.8 ​± ​1.5	85%	NA	CaCO_3_ – OFS (45.8% Lisinopril – Furosemide (7.7%) Captopril – Furosemide (6.6%) Captopril – Spironolactone (6.6%) OFS – Omeprazole/Pantoprazole (5.1%)	0.4%

Schmidt et al. (2019)	Germany	Prospective observational study	5217	60.1 ​± ​12.0	G5 0.0% (G4 3.3%)	8 (median n.; range 0–27)	80%	Simvastatin (38.33%) Aspirin (32%) Ramipril (31%) Allopurinol (30%) Torasemide (28%)	NA. *This study was not specifically aimed at detecting DDI, but at analyzing the burden of polypharmacy in CKD patients. It is here reported as a standard of high-quality evidence*	NA

Santos-Díaz et al. (2020)	Spain	Observational cross-sectional study	122	77.1 ​± ​10.4	4.5%	8.6 ​± ​3.4	73.77%	Omeprazole (30.6%) Acetaminophen (30.6%) Aspirin (26.1%) Bisprolol (25.2%) Furosemide (22.5%)	Acenocoumarol – Omeprazole (1.1%) OFS – Omeprazole (1%) Metformin – Aspirin (1%) Levothyroxine – Omeprazole (1%)	11.4%

Sommer et al. (2020)	Germany	Retrospective observational study	200	78 (IQR 68–85)	33.5% with G5/G5	11 (Median IQR 8-13)	97%	NA	NA	22.56%

ESKD: end stage kidney disease; SD: standard deviation; IQR: Interquartile range; NA: not applicable.

In the prospective observational German CKD (GCKD) study, Schmidt et al. studied 5217 patients with an age ranging between 18 and 74 years, with moderately severe CKD (Schmidt et al., 2019). Self-reported data on medication use were assessed at baseline and after 4 years of follow-up. It was reported that increasing CKD stage, age and body mass index, diabetes mellitus, CVD and a history of smoking were significantly associated with both the prevalence of polypharmacy and its maintenance during the follow up. Diabetes mellitus was also significantly associated with the initiation of polypharmacy.

With respect to medication prevalence in CKD patients, contrasting data are present suggesting that the prescription protocol likely changes over time.

For instance, differently from the findings obtained in the German CKD study, Bailie et al. showed that HMG-CoA reductase inhibitors were only received by 16% of patients (versus 50% in GCKD study) (Bailie et al., 2005).

Laville et al. found a similar medication prevalence in the French Chronic Kidney Disease-Renal Epidemiology and Information Network cohort study (Laville et al., 2018). In this representative, prospective cohort study that enrolled 3033 patients with a mean age of 69 years, with a confirmed diagnosis of CKD stages 3–5 and no previous chronic dialysis or kidney transplantation, the median number of prescribed medications per patient was 8 and it was positively correlated with the severity of CKD. Importantly, the authors were able to highlight inappropriate prescriptions (i.e. contraindicated or inappropriately high dose drug), in a high proportion of the study participants (52%), suggesting the complexity of drug prescription in CKD, given the many different treatment guidelines, the various equations used to measure kidney function, and the pharmacokinetic alteration in renal drug elimination due the decline in GFR.

Secora et al. quantified the medications used by the level of eGFR in 6392 participants aged 65 years or older included in the Atherosclerosis Risk in Communities study (Secora et al., 2018). However, differently from others, the authors reported less frequent use of diabetes medications. Consistently with most observations in this regard, on average, participants with CKD reported more medications and the proportion of participants taking at least one medication for each drug class increased with decreasing eGFR. In addition, the association between higher CKD stage and greater number of medications used persisted in adjusted analyses.

Overall, the previous studies revealed that the mean number of prescribed medications was around 8 with a positive correlation between the number of drugs and the CKD stage. The antihypertensive and lipid-lowering drugs, mainly statins, were the drug classes most commonly used in CKD patients. A non-negligible use of OTC medications was also reported.

## 3 Interaction odds: potential DDI and prevalence of adverse drug reactions

### 3.1 Mechanisms

The alteration in PK and PD parameters often observed in patients with renal insufficiency makes the pharmacological treatment particularly challenging (Matzke et al., 2011). As expected, indeed, the clearance of drugs that fully rely on kidney for elimination is lower, but also important changes were observed in drugs eliminated by intestine and liver. A report from FDA showed that among 37 orally administered drugs in patients with renal failure, 23 were cleared via non-renal pathways and 13 of which were still characterized by an average 1.5-fold increased area under the plasma-concentration-time curve (AUC) in patients with renal disease compared to healthy subjects, suggesting that kidney disease alters many pharmacokinetic and pharmacodynamic properties also of drugs cleared via non-renal route (Zhang et al., 2009). Many mechanisms have been hypothesized, particularly focusing on the effect of CKD on hepatic metabolism.

Cytochrome P450 (CYP) enzymes, responsible for most of the Phase I drug metabolism, are expressed mainly in the liver and intestine but also in the kidney (Yeung et al., 2014). The most likely mechanisms altering drug metabolism in CKD patients are linked to CYP induction and/or inhibition effects, together with the competitive inhibition of CYP enzymes. Nolin and colleagues highlighted a lower expression of CYP in animal models of CKD (Nolin et al., 2008), while an *ex vivo* study carried out on rat hepatocytes showed that serum from patients with ESKD decreased the expression and activity of CYP1A, CYP2C, CYP2D, CYP3A and CYP4A, all of which are involved in the metabolization of xenobiotics (Michaud et al., 2005). Interestingly, only pre-haemodialysis serum caused the decrease of CYP expression, while post-haemodialysis serum showed no effects (Michaud et al., 2008). Cytokines and parathyroid hormone, whose levels are known to be high in CKD, may be responsible for the observed downregulation; accordingly, parathyroidectomy in uremic rats abolished the alteration in CYP transcription and translation (Michaud et al., 2006). Parathyroid hormone effect on CYP expression may involve different molecular mechanisms, including an increase in intracellular calcium levels, increase in cAMP production and activation of NF-kB pathway. The downregulation of CYP expression in response to proinflammatory cytokines, such as IL-6 or TNFα, is well known (Nolin et al., 2008; Michaud et al., 2005, 2006, 2008; Morgan et al., 2008). Other mechanisms leading to an altered drug metabolism in CKD patients are linked to the presence of circulating competitive inhibitors of CYP enzymes, thus downregulating their activity. Yoshitani and colleagues, indeed, demonstrated that uremic serum from rats with renal failure inhibits oxidative metabolism of losartan in rat liver microsomes (Yoshitani et al., 2002). Interestingly, kidney disease and subsequent uremia may also affect drug transporters in the liver, thus impairing the hepatic clearance of drugs and potentially explaining the reduced non-renal clearance observed in CKD patients. In another study, Nolin and colleagues reported that rat hepatocytes exposed to serum from subjects with ESRD showed reduced expression of the Organic Anion-Transporting Polypeptide 1A4 (OATP1A4), together with an increased expression of P-glycoprotein, thus modulating drug disposition (Nolin et al., 2009).

Another aspect that might impact on polypharmacy in patients with CKD is that in a context of multiple organ dysfunction and fluid overload, the volume of distribution of several drugs may be altered (Prowle et al., 2010). In particular, changes in extracellular fluid volume affect mainly hydrophilic compounds or those with low volume of distribution (i.e. <0,6 ​L/kg, such as heparin, warfarin, aminoglycosides, monoclonal antibodies, etc.) (Smith et al., 2015). Thus, these agents should be carefully dosed in a CKD context, particularly if administered in combination, since therapeutic failure or, on the other hand, unpredictable toxicity could occur. Similarly, kidney disease and uremia may affect the ability of plasma protein to bind drugs. Uremic retention solutes and organic wastes can bind to plasma proteins, displace the compound, increase the unbound (free)-fraction of the drug and, consequently, its potential pharmacological effects. These features are of particular importance in polypharmacy patients, possibly leading to a higher interaction potential or direct toxic effects (Olyaei and Steffl, 2011).

### 3.2 DDIs occurrence

The prevalence of potential DDIs in CKD patients undergoing haemodialysis and/or pharmacological treatment has been reported and ranges between 27,5% and 89,1% (Rama et al., 2012; Sgnaolin et al., 2014; Al-Ramahi et al., 2016). This wide range of probability of DDIs is peculiar and many factors may contribute, including pre-existing comorbidities or complications, the number and types of prescribed medications per patient, and also the stage of CKD (Secora et al., 2018).

Potential DDIs can be identified and classified through different methods, including online software, such as LexiComp®, Thomson Reuters Micromedex®, DrugReax® or Medscape drug reference database system®, which provide information about the type, the risk of DDI and its mechanisms, if known, together with recommendations on how to manage DDI. Medscape software, in particular, classifies DDIs in 5 categories based on their level of clinical significance (Fasipe et al., 2018):

- Type A: no known interactions
- Type B: minor or mild interactions. The concomitant use has little or no evidence of clinical concerns
- Type C: modest or significant interactions. The concomitant use requires an appropriate monitoring plan to recognize potentially harmful effects
- Type D: major and serious interactions. The concomitant use should be critically evaluated
- Type X: contraindication. Drugs may interact each other in a clinically significant manner.

Previous studies conducted upon cohorts of patients with CKD on a polytherapy regimen reported that the vast majority of the observed clinically significant DDIs were of moderate severity (type C), followed by mild or minor (type B) DDIs; while major (type D) DDIs were reported as rare as well as type X DDI, that accounted for about 0.1–1% of all DDIs observed (Sgnaolin et al., 2014; Marquito et al., 2014; Hegde et al., 2015; Fasipe et al., 2017a).

Potential DDIs can be further classified into pharmacokinetic, if drug disposition is altered by the coadministration of another drug, affecting thus absorption, distribution, plasmatic binding to proteins, metabolism and excretion, or pharmacodynamic, if the drug effect is altered at the site of action by the presence of a second drug, thus affecting many physiological mechanisms. Finally, pharmaceuticals interactions are frequent, although under-recognized, and occur if more than one drug is administered intravenously at the same time (Hill et al., 2016; Maison et al., 2019). The injected compounds may react and show physicochemical incompatibility within the infused solution, leading to drug inactivity, catheter occlusion, embolism or inflammatory reactions.

Several studies analysed the pattern of potential DDIs in patients with CKD, highlighting the most relevant and common ones (Table 2).

One of the most frequently reported DDIs is between the coadministration of oral CaCO_3_ and oral ferrous sulphate (OFS): this type B and C pharmacokinetic interaction occurs as theintestinal absorption of OFS may be reduced when CaCO_3_ is co-administrated, since CaCO_3_ increases the gastrointestinal pH, leading to a reduced efficacy of OFS treatment. The prevalence of this DDI is extremely heterogeneous, ranging from 45.8% (Okoro and Farate, 2019) to a lower incidence (5.5%-9,9% (Sgnaolin et al., 2014; Hegde et al., 2015; Fasipe et al., 2017a; Fasipe et al., 2017b)).

CaCO_3_ is also reported to interact with amlodipine in many studies, leading to a type C pharmacodynamic interaction: calcium salts, indeed, are known to decrease the effect of calcium channel blockers, thus resulting in hypertension. CaCO_3_ – Amlodipine interaction has been reported as extremely frequent by Al-Ramahi and colleagues (41.5% in patients with CKD under a haemodialysis regimen (Al-Ramahi et al., 2016)), while other studies reported a lower, but significant prevalence (Hegde et al., 2015; Fasipe et al., 2017a, 2017b; Adibe et al., 2017).

The simultaneous treatment with furosemide and ACE inhibitors as lisinopril, captopril and enalapril has been reported in several works to induce severe postural hypotension due to excessive vasodilation and relative intravascular volume depletion and renal insufficiency as a result of low perfusion. This adverse effect occurs especially after the administration of the first dose; the frequency varies depending on the grade of renal impairment and the type of ACE inhibitor administered: furosemide-lisinopril interaction is reported with a frequency of approximately 7–9% (Saleem et al., 2017; Fasipe et al., 2017a; Okoro and Farate, 2019; Adibe et al., 2017), furosemide-enalapril interaction was observed with a frequency of about 5–6% (Marquito et al., 2014; Saleem et al., 2017), while furosemide-captopril interaction occurs with a frequency of approximately 4–6% (Marquito et al., 2014; Saleem et al., 2017; Okoro and Farate, 2019).

OFS has been reported to interact with proton pump inhibitors (PPIs) as omeprazole and pantoprazole in several studies, even if with a relatively low frequency (1–5% (Hegde et al., 2015; Saleem et al., 2017; Okoro and Farate, 2019; Santos-Díaz et al., 2020)). This moderate, type B/C pharmacokinetic interaction rapidly occurs as proton pump inhibitors lead to an increase in gastric pH, thus limiting the absorption of OFS and resulting in a reduced non-heme iron bioavailability.

Furosemide is reported to moderately interact with aspirin in 3 previous studies with a frequency of 4,5–7,9%. Clinical evidence suggest that their concomitant administration results in reduced diuretic and anti-hypertensive effect of furosemide, thus needing to monitor diuresis and creatinine clearance (Marquito et al., 2014; Al-Ramahi et al., 2016; Adibe et al., 2017). The molecular mechanisms underlying this interaction may be related to the well-known effect of cyclooxygenase inhibitors (aspirin and other non-steroidal anti-inflammatory drugs, NSAIDs) on the kidney. In this compartment, NSAIDs counteract the renoprotective actions of the prostaglandins, thus impairing renal blood flow, glomerular filtration rate and natriuresis. Since prostaglandin production is increased in CKD as a mechanism to improve the organ perfusion, the interaction at this level may be particularly relevant (Baker and Perazella, 2020).

In two different studies, Fasipe and colleagues reported a type C, pharmacokinetic interaction between α-Calcidol and CaCO_3_ in about 3% of patients with CKD, resulting in hypercalcemia (Fasipe et al., 2017a, 2017b).

As reported before, the prevalence of type X interaction in extremely low; Saleem and colleagues and Fasipe and colleagues, however, reported some cases of coadministration of calcium glucoronate and ceftriaxone in patients with CKD (Saleem et al., 2017; Fasipe et al., 2018). This pharmaceutical interaction occurs as the intravenous administration of calcium-containing solution, including also Hartmann’s solution and Ringer’s lactate, together with intravenous ceftriaxone is associated with a high potential risk of fatal particulate ceftriaxone-calcium complex precipitates, that deposit into heart, lungs and kidneys, thus compromising their function. Hence, the administration of any intravenous calcium-containing solution and intravenous ceftriaxone must be separated by at least 48 ​h.

As reported in the previous paragraph, lipid-lowering drugs as statins are medications commonly used in CKD patients. Fasipe and colleagues reported that atorvastatin interacts with ritonavir, an antiretroviral drug used in HIV-positive patients. This type D, pharmacokinetic interaction occurs as ritonavir inhibits the membrane influx transporters OATP1B1, that belongs to the superfamily of solute-linked carriers (SLCO)21A, ATP-independent polypeptides, resulting in an increased myotoxicity of atorvastatin (Fasipe et al., 2018). Moreover, some statins are administered as inactive lactone prodrugs (e.g., simvastatin) or are in equilibrium between the lactone or acidic form in plasma and tissues, strongly depending on intestinal and hepatic CYP3A4 for their metabolism (Corsini and Bellosta, 2008). Since ritonavir potently inhibits this CYP, plasma statin concentration may rise with subsequent increased risk of myalgia, pigmenturia and rhabdomyolysis, worsening the renal impairment (Kiser et al., 2008).

A very recent retrospective observational study by Sommer and colleagues (Sommer et al., 2020) analysed the most prevalent medication mistakes due to multiple DDIs in a cohort of 200 elderly patients with stage 3,4,5 of CKD receiving polypharmacy, with the aim of improving medication safety. Interestingly, the study focused primarily on the potential pharmacodynamic interactions. By including patients exhibiting fluctuations in eGFR levels, they identified potentially inappropriate prescriptions related to CKD in 41.5% of patients. Moreover, they found that 29.5% of patients were at increased risk of corrected QT (QTc)-interval prolongation related to medication dosage. Two-thirds of QTc-interval prolongation cases occurred under prescription of amiodarone, citalopram and ciprofloxacin, that were the most commonly prescribed drugs from the ‘known’ risk of Torsades de Pointes (TdP) category. 8.0% of patients received a regimen of 4–6 drugs with potassium enhancing side effects and 75% of them developed hyperkalaemia. Finally, 8.5% of the cohort population received a combination of 3 drugs interfering with haemostasis (i.e., dual antiplatelet therapy plus warfarin) and 87,5% of them developed bleeding complications, including two fatal events.

Finally, in a polypharmacy regimen, some rare but usually severe DDIs were reported in CKD patients taking central nervous system (CNS) acting drugs. In this regard, the prescription of the calcium channel blockers nifedipine and amlodipine in patients treated with an anticonvulsant drugs, e.g. carbamazepine, phenytoin and phenobarbital, led to a severe type X interaction, with a reduction in nifedipine exposure and increased risk of CNS drug toxicity, including ataxia, hyperreflexia, tremor and nystagmus. This absolute contraindication may be likely related to both the induction of nifedipine CYP3A4-mediated metabolism and the reduction of metabolism of phenytoin, carbamazepine or phenobarbital (Marquito et al., 2014; Saleem et al., 2017). Similarly, cotreatment with carbamazepine and omeprazole was reported to induce a moderate adverse outcome (Saleem et al., 2017), as omeprazole is metabolized by CYP3A4 and CYP3C19, while carbamazepine is metabolized by CYP3A4, possibly leading to competitive inhibition of carbamazepine metabolism, significantly altering its PK (increased AUC, prolonged half-life and reduced clearance) (Li et al., 2013). A similar interaction was reported in patients taking carbamazepine along with ciprofloxacin, able to inhibit CYP3A4 isoenzyme, also responsible for carbamazepine metabolism (Shahzadi et al., 2011). Again, Fasipe and colleagues reported a severe pharmacodynamic interaction between α-methyldopa and haloperidol, reducing the clinical efficacy of haloperidol (Fasipe et al., 2018) by acting as a pharmacodynamic antagonist in the chemoreceptor trigger zone (CTZ) site and the mesocorticolimbic system D_2_ receptors, respectively (Markowitz et al., 1995). The same study (Fasipe et al., 2018) reported that phenobarbital when associated with cyclosporin can trigger a severe pharmacokinetic interaction, as phenobarbital, a strong CYP3A and GlycoproteinP (P-gp) inducer, decreases blood concentration of cyclosporin.

Similarly, the association between the antimalaria drugs lumefantrine and promethazine is linked to a major pharmacodynamic interaction by increasing QT interval, possibly leading to cardiotoxicity (Fasipe et al., 2018). Finally, a recent study (Santos-Díaz et al., 2020) identified among rare type X interactions in CKD patients, the coadministration of amitriptyline and aclidinium, as the latter may enhance the anticholinergic effect of amitriptyline (Ajimura et al., 2018).

## 4 Clinical significance and perspectives

Polypharmacy is usually defined as the concomitant use of five or more different medications, although there is currently no official consensus on the cut-off values (Gnjidic et al., 2012; Masnoon et al., 2017). The number of drugs prescribed according to evidence-based guidelines may be high because of multimorbidity and results in complex therapeutic regimens. Kidney disease patients and older people in general may be particularly exposed to this burden, which is linked to an increased risk of DDIs and adverse drug-related events (Sommer et al., 2020). Despite these recognized high-risk conditions, scientific literature on this topic is still scarce. Surprisingly, systematic reviews and meta-analyses covering the DDIs potential in polypharmacy patients affected by renal impairment are completely lacking. To the best of our knowledge, considering population, sample size and study design, only two observational studies provided high-quality evidence about the pattern of medication used in CKD patients (Schmidt et al., 2019) and multiple drug interactions in patients with stage 3, 4 and 5/5D (i.e., undergoing haemodialysis) CKD, also investigating their clinical relevance (Sommer et al., 2020). All the other studies are far less exhaustive and mainly oriented to study the effects of renal impairment on single drug classes. Several other flaws limit the transferability of the current evidence to the routine clinical practice, such as the vast prevalence of retrospective or cross-sectional studies carried out only in selected geographical regions, thus introducing bias caused by the different ethnical, cultural and economic issues (Table 1). In addition, the clinical condition most frequently studied was CKD, while much less attention was paid to other renal diseases, such as AKI, or renal transplantation. The population varies across all the studies and often it is limited to patients receiving regular care by nephrologists, or conversely quite heterogeneous (i.e., hospitalized, critically ill, older patients, etc.). A further complication may come from OTC, supplements, herbal or alternative medications, the use of which is not always considered or is sometimes unknown. Finally, no standardized algorithms exist for DDI determination, and each study applied a slightly different strategy.

As described above in this review, several mechanisms may account for potential DDIs occurrence. The awareness of these concepts may help to reduce the under- or, on the contrary, overestimation of drug combination risk in clinical practice (De Oliveira et al., 2021). For many CKD patients on polypharmacy, reducing the risk of DDIs may not be possible and clinicians may only try to manage that risk to offer the patients the safest treatment option. As an example, the risk of low iron absorption during concomitant OFS and PPIs therapy may be mitigated by recommending the lowest effective dose, the shortest treatment time, supplementation with other iron absorption facilitators (e.g. vitamin C), or a switch to other gastric acid secretion inhibitors, such as the histamine-2-receptor antagonists (McCarthy, 2019; Lam et al., 2017). Other common interactions in this clinical context may occur between calcium supplements and amlodipine, aspirin and furosemide, furosemide and ACE inhibitors, and atorvastatin and ritonavir, respectively. Since CaCO_3_ may antagonize and decrease the vasodilator effect of amlodipine on the small arteries, a frequent blood pressure monitoring may be done for the control of any unwanted reduction of the antihypertensive effect (Fasipe et al., 2017a, 2018; Adibe et al., 2017). Monitoring the body fluid status (e.g. body weight, jugular venous pressure assessment, or spot urine sample (Testani et al., 2016)) may allow managing the poor loop diuretic natriuretic response in patients taking aspirin (or other NSAIDs), thus reducing the risk of fluid overload (Fasipe et al., 2018; Adibe et al., 2017). Moreover, blood pressure and body fluid monitoring may be useful in patients taking loop diuretics and ACE inhibitors, thus preventing the occurrence of severe hypotension. Finally, the risk of myotoxicity and kidney complication in patients taking atorvastatin and ritonavir (or other strong CYP3A4 inhibitors, such as clarithromycin, etc.) could be managed by monitoring serum creatine kinase (CK) level, or by switching to a statin less dependent on CYP3A4 metabolism, such as rosuvastatin (Corsini and Bellosta, 2008; Stroes et al., 2015; Toth et al., 2018).

In summary, no good-quality evidence has been provided so far to guide clinical practice in such populations and the individual physician’s decision is frequently the predominant factor influencing initiation of therapy. One of the key challenge in obtaining appropriate polypharmacy and reducing the risk of DDIs is ensuring that prescribing is evidence-based, also considering that patients with CKD are underrepresented in clinical trials (Parker and Wong, 2019). Therefore, it is of paramount importance to design and conduct *ad hoc* studies, preferably involving a renal multidisciplinary team. There is some evidence that involving clinical pharmacologists or clinical pharmacists in the management of polypharmacy patients significantly improved the capability to uncover, prevent or manage the risk of DDIs (Al Raiisi et al., 2019; Hawley et al., 2019). The implementation of such professional figures in CKD patient care is beneficial in supporting clinicians and benefit patients, leading to increased medication knowledge, decreased length and rates of hospitalization, and significantly improved the quality of life in renal patients even at the latest stage of disease (Stemer and Lemmens-Gruber, 2011; Salgado et al., 2013; Sathvik et al., 2007; PAI et al., 2009; Pai et al., 2009).

## 5 Conclusions

CKD is the progressive decline in renal function over time. These patients require several medications to treat a variety of comorbid conditions. Despite a clinical rationale for the use of most, if not all, of the medications prescribed, the overall risk to benefit ratio in one individual should be carefully weighed in the context of polypharmacy. Overall, the number of drugs prescribed per patient averages eight and the most commonly used are drugs for diabetes, hypertension, and cardiovascular disease. Half of the patients received at least one inappropriate drug prescription. Importantly, renal insufficiency is accompanied by alterations in the pharmacodynamic/pharmacokinetic parameters, placing CKD patients at further increased risk of changes in drug accumulation, disposal and unwanted effects. In polypharmacy patients, these events lead to different DDIs, possibly causing serious medical problems or even a major clinical effort to better manage unavoidable DDIs. Very few studies addressed this problem so far and comprehensive guidelines for clinicians are still lacking. High-quality studies are warranted in the near future, in order to provide substantial evidence and support clinical decisions in this particular field.

## CRediT authorship contribution statement

**Bianca Papotti:** Resources, Writing – original draft, Writing – review & editing. **Cinzia Marchi:** Writing – original draft, Writing – review & editing. **Maria Pia Adorni:** Writing – original draft, Writing – review & editing, Supervision. **Francesco Potì:** Resources, Writing – original draft, Writing – review & editing, Supervision.

## Declaration of competing interest

The authors declare that they have no known competing financial interests or personal relationships that could have appeared to influence the work reported in this paper.
